# Physiological and Molecular Mechanisms of Lepidopteran Insects: Genomic Insights and Applications of Genome Editing for Future Research

**DOI:** 10.3390/ijms252212360

**Published:** 2024-11-18

**Authors:** Dongsheng Niu, Qing Zhao, Linbo Xu, Kejian Lin

**Affiliations:** 1 Institute of Grassland Research, Chinese Academy of Agricultural Sciences, Hohhot 010000, China; niudongsheng@caas.cn (D.N.); zhaoqing01@caas.cn (Q.Z.); 2 Inner Mongolia-CABI Joint Laboratory for Grassland Protection and Sustainable Utilization, Chinese Academy of Agricultural Sciences, Hohhot 010000, China; 3 Key Laboratory of Biohazard Monitoring, Green Prevention and Control for Artificial Grassland, Ministry of Agriculture and Rural Affairs, Chinese Academy of Agricultural Sciences, Hohhot 010000, China; 4 Inner Mongolia Key Laboratory of Grassland Protection Ecology, Chinese Academy of Agricultural Sciences, Hohhot 010000, China

**Keywords:** lepidopteran insects, insecticide resistance, functional genomics, gene editing, CRISPR/Cas9, pest management, genomic data, sustainable agriculture

## Abstract

Lepidopteran insects are a major threat to global agriculture, causing significant crop losses and economic damage. Traditional pest control methods are becoming less effective due to the rapid evolution of insecticide resistance. This study explores the current status and genomic characteristics of 1315 Lepidopteran records, alongside an overview of relevant research, utilizing advanced functional genomics techniques, including RNA-seq and CRISPR/Cas9 gene-editing technologies to uncover the molecular mechanisms underlying insecticide resistance. Our genomic analysis revealed significant variability in genome size, assembly quality, and chromosome number, which may influence species’ biology and resistance mechanisms. We identified key resistance-associated genes and pathways, including detoxification and metabolic pathways, which help these insects evade chemical control. By employing CRISPR/Cas9 gene-editing techniques, we directly manipulated resistance-associated genes to confirm their roles in resistance, demonstrating their potential for targeted interventions in pest management. These findings emphasize the value of integrating genomic data into the development of effective and sustainable pest control strategies, reducing reliance on chemical insecticides and promoting environmentally friendly integrated pest management (IPM) approaches. Our study highlights the critical role of functional genomics in IPM and its potential to provide long-term solutions to the growing challenge of Lepidopteran resistance.

## 1. Introduction

Lepidopteran insects, comprising around 160,000 species including moths, butterflies, and skippers, represent the second-largest order of insects, with moths constituting 90% of this group [1,2]. Many Lepidopteran species, such as the cotton bollworm (*Helicoverpa armigera*) and the diamondback moth (*Plutella xylostella*), are notorious pests that cause extensive crop damage, causing significant crop damage and posing risks to food security [3,4]. Lepidopteran insects are challenging to control due to their high reproductive rates, mobility, and notable ability to develop resistance to pesticides, which diminishes the effectiveness of conventional strategies like chemical insecticides, genetically modified crops, and insect viruses [5,6,7].

The development of insecticide resistance in Lepidopteran pests is a complex, multifaceted process driven by genetic mutations [8], behavioral changes [9], and physiological adaptations [10]. These adaptations enable Lepidopteran insects to survive exposure to chemical treatments. Key resistance mechanisms include enhanced metabolic detoxification via metabolic enzymes, alterations in target-site sensitivity, reduced penetration or increased excretion of chemicals, and behavioral modifications that minimize insecticide exposure [9]. As resistance becomes more prevalent, there is an urgent need for innovative strategies that can provide more sustainable solutions to manage these pests effectively [6,11].

Recent advances in genomic technologies, including RNA-seq, whole genome sequencing (WGS), and CRISPR/Cas9 gene-editing, have provided powerful tools for studying the genetic basis of resistance [12,13]. The availability of complete genomic sequences from key species like *Bombyx mori* has opened new avenues for investigating the genetic foundations of resistance [14,15]. However, despite these advancements, significant challenges remain in translating gene-editing breakthroughs to practical pest management applications. Issues such as potential off-target effects, regulatory hurdles, and the ecological risks associated with releasing gene-edited organisms into the environment must be carefully considered [16,17]. Additionally, the high genetic diversity and adaptive capabilities of pest populations complicate efforts to achieve durable resistance management. By integrating genomic data with advanced gene-editing techniques, researchers are now poised to explore new dimensions of pest biology, paving the way for innovative pest management strategies [18,19].

This review aimed to synthesize the current understanding of the molecular mechanisms behind insecticide resistance in Lepidopteran pests, focusing on the role of functional genomics and gene-editing technologies in uncovering these mechanisms. Additionally, we addressed the challenges and limitations of applying these tools in pest management, such as the complexities of gene-editing delivery methods and the need for sustainable, environmentally friendly solutions. By highlighting the potential of these tools, this review underscores their transformative role in advancing integrated pest management (IPM) and provides a roadmap for future research that could lead to more effective pest control strategies.

## 2. Lepidoptera Genome Data and Literature Retrieval Strategies

We conducted a search in the National Center for Biotechnology Information (NCBI) database (https://www.ncbi.nlm.nih.gov/datasets/genome/ (accessed on 28 September 2024)) using the keyword “Lepidoptera”, retrieving 2562 records as of 28 September 2024. Following a rigorous manual curation and verification process, 1315 records were selected for a further year-by-year heatmap analysis (see Table S1). For duplicate species, we selected the most recent genomic data based on the assembly level (contig, scaffold, chromosome, complete). We retained the datasets with the highest assembly quality and removed the lower-level assemblies. To investigate genomic divergence across Lepidopteran taxonomic families, we focused on chromosome-level reference genomes from 594 taxa, representing 45 out of 50 recognized superfamilies (90%). Principal component analysis (PCA) was then performed on these 599 taxa, using 11 genome assembly metrics: genome size, total ungapped length, number of chromosomes, number of scaffolds, scaffold N50, scaffold L50, number of contigs, contig N50, contig L50, GC content, and genome coverage. Both heatmap and PCA visualizations were generated using R software (version 4.3.3).

The genome data we collected are essential for understanding the genetic basis of insecticide resistance in Lepidoptera. By selecting high-quality genome assemblies from a broad range of taxa, this dataset provides valuable insights into the genetic diversity and evolutionary patterns across different species. These genomic resources help identify genes and pathways involved in resistance, offering new directions for pest control strategies.

In parallel, we conducted a comprehensive literature search in the Web of Science (WOS) and Scopus databases, applying Boolean operators to focus on research related to insecticide resistance and management in Lepidoptera, with a special emphasis on RNAi and gene-editing technologies (see Table 1). Reviews, commentaries, and conference proceedings were excluded to focus on primary research articles that provide original, peer-reviewed data and experimental results. There were no temporal restrictions on the literature search, and only articles published in English were considered. The inclusion criteria were as follows: (1) studies must focus on Lepidoptera species relevant to insecticide resistance; (2) research must involve RNAi, TALENs, or CRISPR/Cas9 technologies specifically targeting genes associated with insecticide resistance; and (3) studies lacking a focus on insecticide resistance were excluded. The selected literature was imported into Zotero for deduplication, followed by a thorough manual screening, resulting in 150 relevant articles for in-depth review.

For the literature of RNAi application in resistance-related genes in Lepidopteran insects, we assigned fixed impact strength values to different effect descriptions, employed regular expressions to match textual descriptions, and then computed the impact strength according to Table 2. The impact strength of target genes across different developmental stages of insects was visualized using the ggplot2 package. The literature with an impact strength of ≥10 was selected for further review.

## 3. Recent Advances in Lepidopteran Genomics

Lepidoptera, a group that includes butterflies and moths, represent approximately one-tenth of all described species on Earth, with most possessing a standard chromosome number of 31 [20]. Despite their diversity, genomic research on Lepidopterans has been challenging due to their large and complex genomes. However, recent breakthroughs in genomics have significantly advanced our understanding of the molecular biology and physiology of these insects. The development of high-throughput sequencing technologies has facilitated the assembly of high-quality genomes for several key Lepidopteran species, unveiling critical genes involved in insecticide resistance, development, and reproduction.

### 3.1. Current Status of Lepidopteran Genomic Research

**Figure 1:**
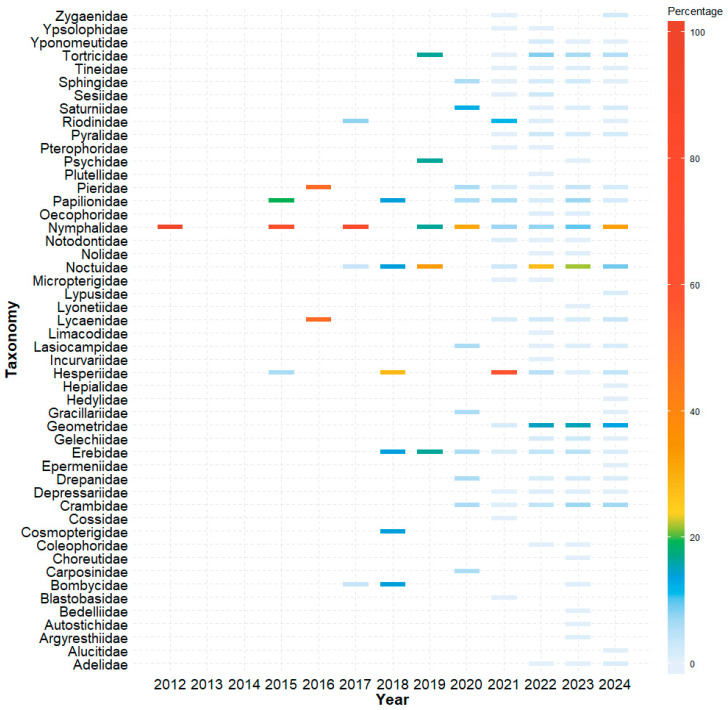
Annual heatmap of Lepidoptera genome. The horizontal axis represents the release years of the sequenced genomes, while the vertical axis denotes different families within Lepidoptera. The color blocks indicate the proportion of genomes released by each family in a given year. The colors range from blue to red, with red indicating a higher percentage of genome releases within a family during a specific year. This visualization provides an overview of genome sequencing efforts across different Lepidopteran families over time to identify peak sequencing periods and family-specific trends.

The i5k initiative, aimed at sequencing 5000 arthropod genomes, has accelerated Lepidopteran genomics research. Since the sequencing of the first Lepidopteran pest genome—*Plutella xylostella* (diamondback moth) in 2013—the number of Lepidopteran genomes has steadily increased [21,22,23]. As of 28 September 2024, the genomes of 1315 Lepidopteran species were sequenced, primarily from the families Nymphalidae, Noctuidae, and Geometridae (Figure 1). Despite this progress, only 599 genomes have achieved chromosome-level assembly. Of these, 4 genomes are classified as complete, 138 at the scaffold level and 578 at the contig level. This highlights the ongoing challenges in obtaining fully assembled genomes for many Lepidopteran species, particularly those with larger and more complex genomic structures (see Table S1).

### 3.2. Genomic Characteristics of Lepidopteran Insects

**Figure 2:**
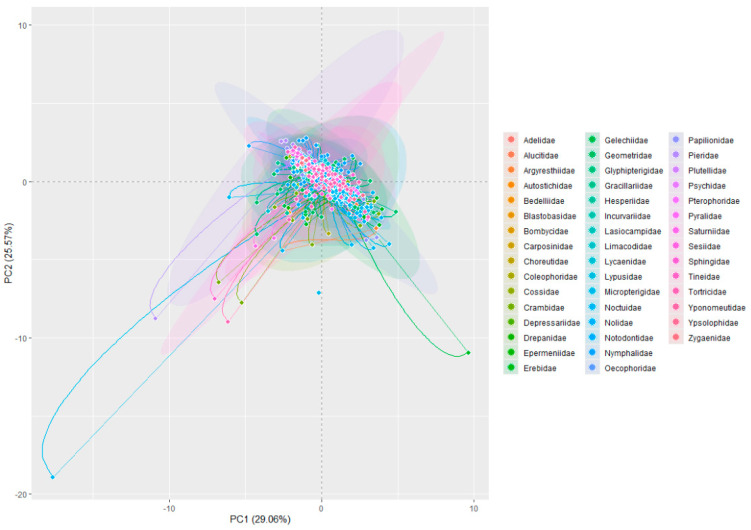
PCA biplot of Lepidoptera genome traits showing variability across major families. Each colored dot in the figure represents different Lepidopteran species’ samples, with colors and shapes corresponding to different taxonomic families. The ellipses represent 95% confidence intervals for each family, indicating intra-family variation. Lines on the plot represent genomic variables, such as genome size and chromosome number. The angle between lines reflects correlations among variables, and line length indicates the standard deviation. Distances between points represent similarities between species, and ellipses denote the 95% confidence intervals.

In a principal component analysis (PCA) of 599 chromosome-level genomes from Lepidoptera species, the first two principal components (PC1 and PC2) accounted for 29.06% and 25.57% of the variance, respectively, totaling 54.63% of the total variation (Figure 2). While this percentage is moderate, it highlights key trends in the dataset. The taxonomic groups displayed varying degrees of overlap and separation across PC1 and PC2, with certain families showing distinct divergence, potentially due to differences in their morphological or ecological traits. Most families were clustered near the center of the plot, indicating considerable overlap and suggesting significant trait similarities among these taxa. However, some groups, particularly those distributed toward the outer sections of the plot, exhibited greater separation, indicating unique characteristics in either PC1 or PC2. This suggests notable differences in morphological or ecological adaptations across Lepidopteran families.

The PCA further revealed significant differences in genome size, assembly quality, and chromosome number among species. PC1 (30.49% of the variance) was mainly influenced by genome size and total ungapped length, with larger genomes generally showing poorer assembly quality (as indicated by lower N50 and L50 values). PC2 (28.12% of the variance) highlighted the contrast between species with more chromosomes and those with higher numbers of scaffolds and contigs. Overall, larger genomes tended to exhibit lower assembly quality, while species with more chromosomes had fewer scaffolds and contigs. These results provide valuable insights into the genomic diversity across Lepidopteran species, shedding light on key genomic attributes that may influence their biology and evolution.

### 3.3. Genomic Specificity and Its Role in Resistance Adaptation

The genomic diversity provides a foundation for understanding the mechanisms of insecticide resistance [24]. Variations in genome size, chromosome structure, and assembly quality among Lepidopteran species can impact gene expression and genetic adaptability, which are directly relevant to resistance traits [25,26]. For instance, unique genomic features—such as gene duplications and structural variants—may facilitate rapid adaptation to insecticides, enabling certain species to better withstand chemical control measures [27].

Genomic specificity, particularly in regions linked to detoxification enzymes and target-site mutations, plays a pivotal role in resistance development [28]. These unique genomic attributes can aid in identifying molecular targets for novel pest control strategies and in predicting resistance evolution. Therefore, studying Lepidopteran genomic characteristics provides not only evolutionary insights but also a basis for enhancing pest resistance management.

## 4. Overview of Functional Genomics Technologies

### 4.1. Applications of Genomics and Functional Genomics in Resistance Adaptation

Functional genomics leverages insights and tools generated by structural genomics to comprehensively analyze gene functions from a genomic or systems perspective. This field employs high-throughput techniques such as RNA sequencing, microarray analysis, and proteomics [29], shifting biological research from the study of individual genes or proteins to a systemic study of multiple genes and proteins, with a focus on dynamic biological functions rather than static genomic sequences. Core research areas in functional genomics include gene function discovery, gene expression analysis, and mutation detection, all of which aim to elucidate interactions between genes and biological systems, thereby bridging the gap between genomic sequences and phenotypic expression.

**Figure 3:**
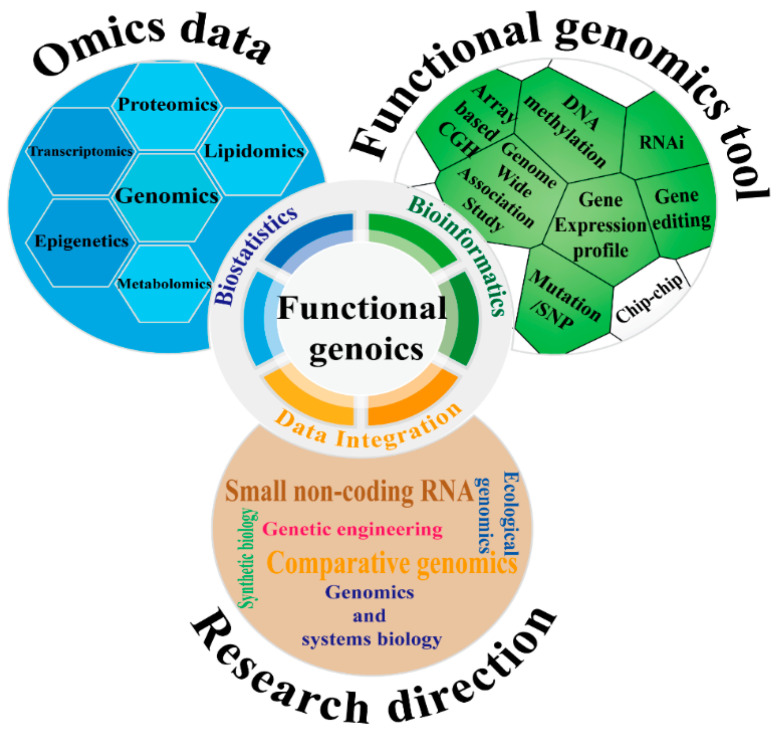
Comprehensive overview of key research techniques and directions in insect functional genomics. Blue blocks represent Omics data-related content, green blocks indicate tools related to functional genomics, and brown blocks denote research directions.

Figure 3 provides a comprehensive overview of key data types, tools, and research directions in functional genomics. The omics data layer encompasses various omics technologies, including transcriptomics [30], proteomics [31], metabolomics [32], and epigenetics [33,34], which offer extensive information on genes and molecules. The functional genomics tools layer lists a range of techniques for gene function analysis, such as DNA methylation analysis, RNA interference (RNAi), and gene editing, which support the in-depth exploration of gene functions. Lastly, the research directions layer highlights emerging fields such as small non-coding RNA research [35], genomics and systems biology [36], ecological genomics [37], comparative genomics [38], synthetic biology [39], genetic engineering [40], and bioinformatics [41]. Together, these elements are integrated through bioinformatics and biostatistics to provide a solid foundation for resistance studies by analyzing complex biological data comprehensively.

Currently, studies are primarily focused on species such as the silkworm [42], honeybee [43], mosquito [44], fly [45], and various agricultural pests [46,47,48,49]. Moreover, clinical and agricultural translational research is applying these findings to practical production, promoting biological control and crop improvement and supporting sustainable agriculture [50].

### 4.2. RNA Interference: Mechanisms and Applications

RNAi is a conserved gene-silencing mechanism found across various organisms, including plants, animals, and microorganisms [51]. RNAi mediates the degradation of messenger RNA (mRNA) sequences via short interfering RNA (siRNA) [52]. Despite its advantages of high specificity, broad-spectrum efficacy, high efficiency, systemic action, and environmental safety, RNAi faces significant challenges in practical pest control applications, especially in terms of delivery and stability in field conditions [53]. Unlike traditional germline transformation methods, RNAi allows the delivery of dsRNA and siRNA through various approaches, though challenges remain in developing effective delivery methods for field applications, especially in pest control [54].

**Figure 4:**
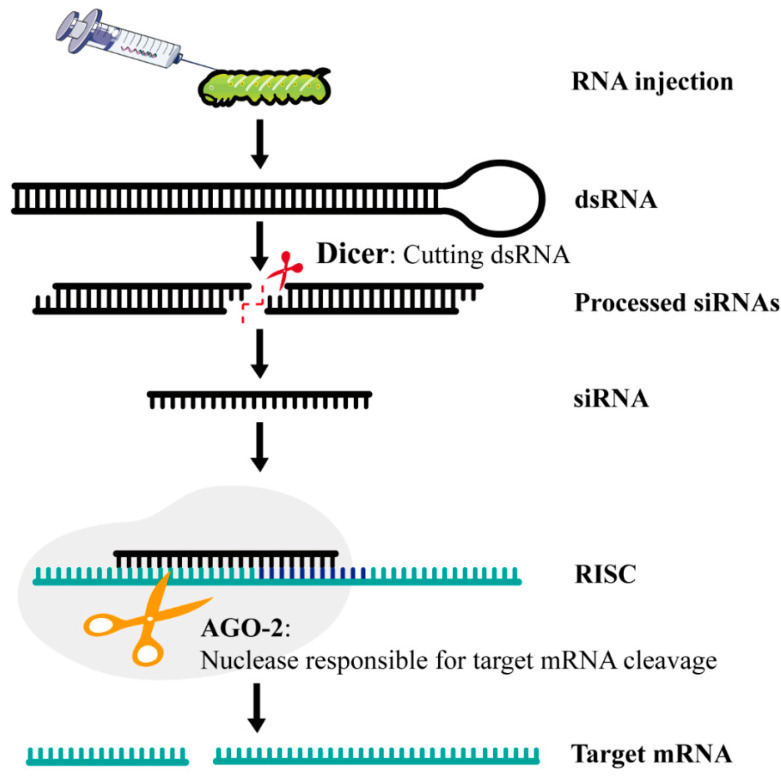
Schematic of delivery system and basic mechanism RNAi in insects. Double-stranded RNA (dsRNA) is cleaved into fragments of around 21 nucleotides (the small interfering RNAs, or siRNAs) by the enzyme Dicer. The siRNAs’ antisense strands couple to the RNA-induced silencing complex (RISC) and convey it to target mRNA, blocking and degrading it.

RNAi can be classified into three main pathways: siRNA (short/small interfering RNA), miRNA (microRNA), and piRNA (PIWI-interacting RNA) [53]. The siRNA pathway involves Dicer cleavage of dsRNA into siRNA fragments, which then integrate into the RNA-induced silencing complex (RISC) to degrade target mRNA (Figure 4) [55,56]. The miRNA, in contrast, prevents mRNA translation by binding to a non-coding region of mRNA, effectively halting protein synthesis [57,58]. The piRNA interacts with Argonaute proteins and silences target RNA through base pairing and subsequent degradation [59,60]. Each of these pathways has distinct roles and mechanisms in gene silencing, with potential applications in pest control. For instance, while siRNA is commonly used for direct gene knockdown, miRNA can be harnessed for regulating broader gene networks, and piRNA has unique roles in transposon silencing, which could be leveraged in genetic control strategies for pests.

### 4.3. Advances in Gene Manipulation Technologies: GAL4/UAS, TALEN, and CRISPR/Cas9

**Figure 5:**
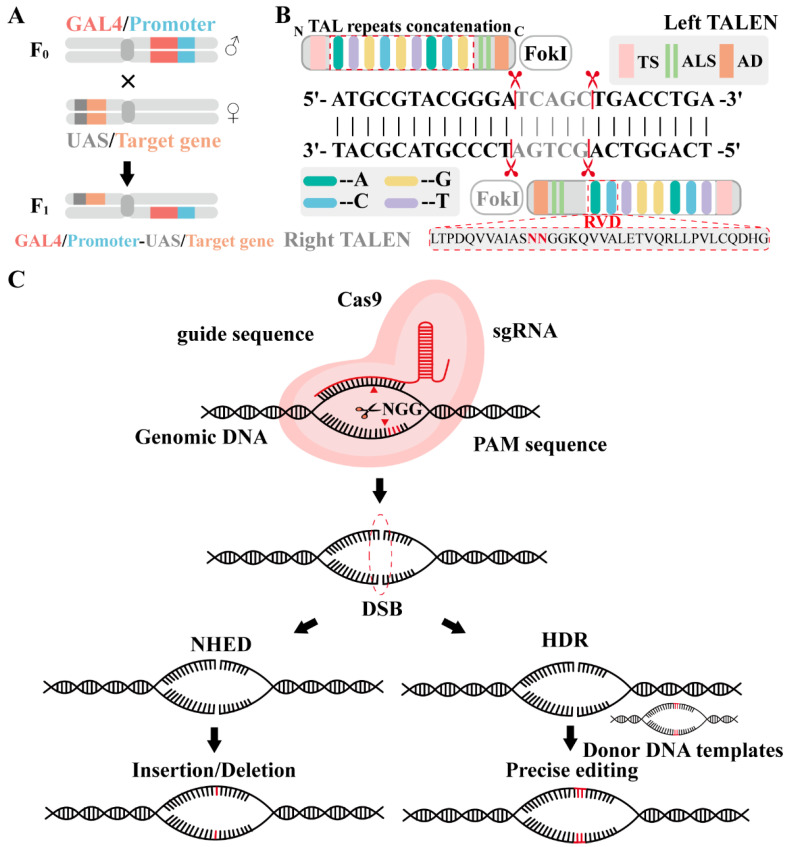
Schematic of gene manipulation technologies: GAL4/UAS, TALEN, and CRISPR/Cas9. (**A**) GAL4/UAS system: in the F_1_ generation, GAL4 from the first transgenic strain binds to UAS in the second strain, activating the target gene. (**B**) TALEs’ system: the TALE system includes both left (black) and right TALEs (gray). TALE proteins are composed of TAL repeats’ concatenation, along with N-terminal and C-terminal regions, a nuclear localization signal (NLS), an activation domain (AD), and a type III secretion signal (TS). These proteins consist of tandem repeats, with each repeat binding to a single DNA base via its repeat-variable diresidues (RVDs). The four primary RVDs—NN, NG, NI, and HD—specifically recognize the bases G, T, A, and C, respectively. (**C**) CRISPR/Cas9 system: Cas9 is guided by a single-guide RNA (sgRNA) to the target DNA sequence, inducing a double-strand break three bases upstream of the PAM (NGG). The break is repaired by non-homologous end joining (NHEJ) or homology-directed repair (HDR), using a donor DNA template for precise modifications.

The GAL4/UAS system is a binary expression system composed of two key elements: the galactose-regulated upstream promoter element 4 (GAL4) and the upstream activation sequence (UAS) (Figure 5A). Both GAL4 and UAS originate from yeast, where they act as regulatory factors involved in galactose metabolism. Specifically, GAL4 binds to UAS, initiating the expression of downstream genes [56]. A major advantage of this system is its flexibility, allowing researchers to express any candidate gene of interest [61]. This expression can be controlled by heat shock protein (HSP) promoters, leveraging their heat sensitivity. By adjusting the temperature and duration of heat exposure, gene expression can be precisely regulated [62].

TALENs technology is a second-generation gene-editing method, originating from the Xanthomonas genus of plant pathogens [63,64]. Structurally similar to zinc-finger nucleases (ZFNs), TALENs function through a distinct mechanism. Each TALEN consists of two domains: the N-terminal transcription activator-like effector (TALE) domain, which binds to a specific DNA sequence, and the C-terminal Fok I nuclease domain, which induces double-strand breaks in the DNA for gene editing [65]. Each TALE protein is composed of three parts: an N-terminal with transport signals, a C-terminal with nuclear localization signals (NLSs) and an acidic transcriptional activation domain (AD), and a central DNA-binding domain (Figure 5B). The DNA-binding domain consists of tandem repeat sequences, where each repeat contains 33–35 highly conserved amino acids. The 12th and 13th amino acids are variable, known as repeat-variable diresidues (RVD), which enable specific recognition of DNA bases [66]. For instance, NI recognizes A, NG recognizes T, HD recognizes C, NK recognizes G, and NN recognizes G or A (Figure 5B) [66,67]. By designing TALE modules with specific RVDs, TALENs can theoretically target any DNA sequence.

The CRISPR/Cas gene-editing technology is recognized as a third-generation gene-editing tool [56]. It is an RNA-guided endonuclease system that originally evolved in bacteria and archaea as an adaptive immune defense against invading foreign nucleic acids [68]. The system consists of two primary components: clustered regularly interspaced short palindromic repeats (CRISPR) and CRISPR-associated proteins (Cas) (Figure 5C) [69]. CRISPR/Cas is classified into two classes and six types: Class 1, which includes multi-subunit effector complexes (Types I, III, IV), and Class 2, which consists of single-protein effectors (Types II, V, VI). The most commonly used systems are Cas9 (Type II), Cas12 (Type V), and Cas13 (Type VI) [70,71]. To enhance gene-editing efficiency, three strategies have been developed: (1) introducing plasmid vectors encoding Cas9 and single-guide RNA (sgRNA) in vitro [72]; (2) injecting plasmid DNA encoding sgRNA into transgenic *Drosophila* [73]; and (3) establishing a transgenic model containing Cas9-sgRNA constructs [74].

While CRISPR/Cas9 has revolutionized gene editing, its application in pest control, particularly in Lepidoptera species, raises ethical and regulatory concerns due to potential ecological risks and gene drive issues. The CRISPR immune defense mechanism consists of three stages (Figure 5C): adaptation, pre-crRNA expression and processing, and interference [56]. During the adaptation phase, when foreign nucleic acids first invade the bacterial genome, the CRISPR system identifies the protospacer adjacent motif (PAM) sequence in the foreign DNA and incorporates approximately 30 nucleotides of the upstream protospacer sequence into the CRISPR array, forming a new spacer and thereby establishing immune memory [75]. In the pre-crRNA synthesis and processing stage, the CRISPR system transcribes pre-crRNA under the guidance of the leader sequence, and these crRNAs are subsequently processed into mature crRNAs by specific Cas proteins. Finally, during the interference phase, mature crRNAs form a complex with Cas proteins, which recognize the protospacer sequence in the foreign nucleic acid through base pairing, guiding Cas proteins to cleave the foreign nucleic acid, effectively preventing further invasion [76].

## 5. Molecular Mechanisms of Insecticide Resistance in Lepidopteran Insects

### 5.1. Evolution and Molecular Basis of Insecticide Resistance: Key Factors Such as Gene Mutations and Metabolic Enzymes

Insecticide resistance in Lepidopteran insects is primarily driven by the extensive use of insecticides, resulting in notable genomic changes. Mechanistically, insecticide resistance can be categorized into four main types: metabolic resistance, target-site resistance, penetration resistance, and behavioral resistance [77]. Among these, metabolic and target-site resistance are considered the most important mechanisms contributing to insect resistance [78].

Metabolic resistance involves the increased ability of insects to detoxify insecticides through the elevated expression or activity of detoxifying enzymes, notably cytochrome P450 (CYP), esterases (EST), and glutathione S-transferases (GST) [79,80]. Cytochrome P450 enzymes, including *CYP6* family genes (e.g., *CYP6A1*, *CYP6A2*), are confirmed or suspected to be linked to insecticide resistance [81,82]. Esterases and GSTs, particularly from the δ and ε families, further contribute to resistance by neutralizing toxic compounds, often via gene amplification or mutation [83]. Additionally, ABC transporters are also involved in detoxifying insecticides by exporting toxins from cells [84].

Target-site resistance occurs through mutations or reduced expression in target molecules that diminish insecticide binding sites, thereby increasing resistance. Key targets include acetylcholinesterase (AChE), sodium ion channels (SC), and γ-aminobutyric acid (GABA) receptors [85]. Mutations in AChE lead to resistance against organophosphates and carbamates [86], while alterations in sodium channels confer resistance to DDT and pyrethroids. Mutations in γ-aminobutyric acid (GABA) disrupt inhibitory neurotransmission, resulting in resistance to insecticides targeting these pathways [87].

### 5.2. RNAi Applications in Targeting Resistance Genes

**Figure 6:**
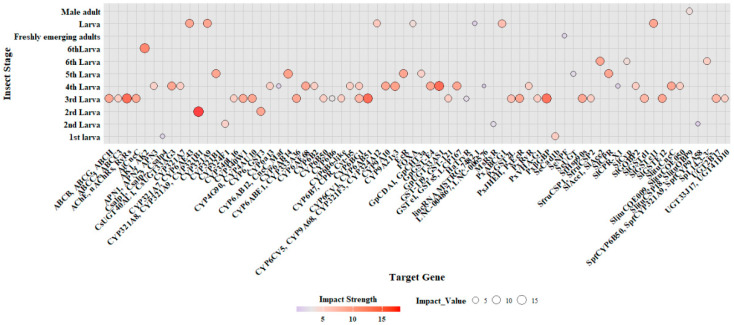
Impact strength of target genes across developmental stages of insects. The *x*-axis represents the target genes, while the *y*-axis shows the developmental stages of the insects. The color intensity of the bubble indicates the impact strength, with darker shades representing stronger impacts.

RNAi is a key technique for studying resistance-related genes in Lepidopteran insects by delivering specific *dsRNA* to silence these targeting genes and assessing the impact on insect survival rates and resistance levels (Table S2). The bubble map in Figure 6 demonstrates the variation in the impact strength of specific target genes across different developmental stages of Lepidopteran insects. Most studies on high-impact genes (impact strength ≥ 10) are concentrated primarily on the third to fifth larval stages (Figure 6 and Table S2).

A variety of genes associated with detoxification and resistance mechanisms were identified, particularly cytochrome P450s (CYPs), ATP-binding cassette (ABC) transport proteins, UDP-glucuronosyltransferases (UGT), and glutathione S-transferases (GST) (Table S2). These genes are essential in enhancing the insect’s resilience to chemical insecticides, with notable examples including *CYP321A8/CYP321A9*/*CYP321B1* [88], *CYP6CV1/CYP6AB51* [89], *ABCB/ABCG/ABCH* [90], *GpUGT33AS1* [91], and *GpGST-E4* [92]. These enzymes play crucial roles in metabolizing chemical insecticide like chlorantraniliprole, tetraniliprole, spinetoram, and emamectin benzoate, thus contributing to resistance [93,94,95]. Additionally, silencing *SpL14-3-3ζ*, *ABCC2/ABCC3*, and *PxβGBP* increased sensitivity to fungal infection [96,97,98]. Silencing these genes disrupts the insect’s ability to resist insecticides, reinforcing their involvement in resistance mechanisms.

The genes involved in the development and growth of the insect were also investigated. Notably, *AK1/AK2* [99] play critical roles in detoxification and maintaining insect viability. Silencing the *CYP6B6* gene in *Helicoverpa armigera* resulted in growth stunting and reduced tolerance to insecticides [100]. Similarly, silencing the *SlCyp* gene in *Spodoptera litura* impacted larval growth and altered phenotypes during pupal and adult stages [101]. These genes are generally associated with growth regulation, metabolic pathways, and endocrine systems, underscoring their importance throughout the insect lifecycle. Moreover, gene silencing can also influence insect reproductive behavior, especially in mating and oviposition. Silencing the *SeNPF* gene in *Spodoptera exigua* reduced mating and oviposition rates, indicating its role in behavior regulation [102]. Such insights open avenues for targeting specific genes to manage pest reproduction and population dynamics.

In summary, this study emphasizes the pivotal roles of genes associated with insecticide resistance, detoxification, and developmental regulation, providing crucial insights into the molecular basis of pest resilience to chemical control. Targeting these genes could significantly enhance pest control strategies, increasing insect susceptibility to insecticides and disrupting physiological functions essential for growth and reproduction. These findings identify promising gene targets for RNAi-based pesticides, advancing the potential for sustainable pest management. However, challenges remain for RNAi applications, particularly in delivery efficiency and species-specific efficacy [103,104], highlighting the need for further research to optimize this approach across diverse agricultural settings.

### 5.3. Advances in TALENs and CRISPR/Cas9 Editing of Resistance Genes

TALENs and CRISPR/Cas9 technologies have revolutionized the study of insecticide resistance in Lepidopteran insects, providing precise gene-editing capabilities to investigate resistance-related genes (Table 3).

Notably, CRISPR/Cas9 editing of the *ABCC2* gene resulted in *Bombyx mori* mutants’ resistant to Cry1Ac toxin, demonstrating the crucial role of *ABCC2* in mediating Cry toxin resistance [105]. Similarly, TALENs-based modification of the *ABCB1* gene in *Bombyx mori* uncovered its function as a receptor for Cry toxins [106]. In *Helicoverpa zea*, mutations in *ABCC2* resulted in a 7.3- to 39.8-fold increase in Cry1Ac resistance, while *HzABCA2* mutations led to a more than 200-fold resistance to Cry2Ab [111,112]. In *Plutella xylostella*, *PxABCC2* and *PxABCC3* were confirmed as critical midgut receptors for Bt Cry1 toxins, further establishing their importance in resistance mechanisms [115]. In *Ostrinia furnacalis*, CRISPR/Cas9 editing of the *OfABCC2* gene identified an 8 bp deletion mutation that conferred Cry1Fa resistance, while disruption of the *OfCad* gene led to moderate Cry1Ac resistance [113,114]. These findings significantly enhance our understanding of the genetic basis of Cry resistance in Lepidopteran pests. In *Spodoptera frugiperda*, *SfABCC2* functioned as a Cry1F receptor, and its disruption conferred resistance to Cry1F toxins [124].

In *Chilo suppressalis*, CRISPR/Cas9-induced mutations in the *RyR* gene conferred resistance to diamide insecticides [107], demonstrating the effectiveness of CRISPR/Cas9 in identifying genetic drivers of resistance across different species. In *Helicoverpa armigera*, CRISPR/Cas9 confirmed *HaCad* as a functional receptor of Cry1Ac, while editing a cluster of nine *P450* genes significantly reduced survival when exposed to host plant chemicals and insecticides [109]. Additionally, mutations in *PgABCA2* increased resistance to Cry2Ab [110], illustrating the complex interplay of multiple mutations in resistance mechanisms.

In *Spodoptera exigua*, mutations in *SeRyR* conferred high resistance to diamide insecticides [117], while knockouts of *SeP-gp* increased susceptibility to abamectin and emamectin benzoate [118]. Mutations in *nAChR α 6* were associated with a dramatic 373-fold resistance to spinosad [119,121], emphasizing the power of CRISPR/Cas9 in isolating the genetic foundations of resistance. Additionally, knockouts of *CYP9A186* restored susceptibility to emamectin benzoate [120], and *CYP9A* was found to detoxify both plant defense compounds and insecticides [122]. In *Spodoptera exigua*, CRISPR/Cas9 editing of *nAChR α 1–α 7* increased resistance to various insecticides, with the *Se α 1* knockout showing enhanced resistance to insecticides compared to the wild-type [123]. The CRISPR/Cas9-based knockout of *Vip3Aa* resulted in high resistance to *Vip3Aa* in fall armyworm and other pests, further solidifying the role of *Vip3Aa* in pest control [125]. Additionally, the knockout of *SfUGT50A15* in *Spodoptera frugiperda* strains led to higher sensitivity to several insecticides than wild-type strains, indicating the involvement of this gene in insecticide detoxification [126].

These findings illustrate the powerful applications of TALENs and CRISPR/Cas9 in dissecting the genetic basis of insecticide resistance, offering insights that could shape more effective pest management strategies. However, CRISPR/Cas9 has limitations, including off-target effects, delivery challenges, and variability in efficacy across pest species, pointing to the need for further refinement in gene-editing applications [17,18].

### 5.4. Genomic Insights into Controlling Insecticide Resistance of Lepidopteran Insects

Insecticide resistance in Lepidopteran insects has evolved due to the selective pressure exerted by extensive pesticide use, making it an inevitable trend in pest populations [127]. The primary goal of resistance research is to delay resistance development, particularly to prevent cross-resistance during pesticide rotation [128]. Current research approaches include measuring indoor toxicity levels, analyzing enzyme activities, and quantifying gene expression, along with cloning insecticide target genes and conducting genetic studies on resistance mechanisms. In the aforementioned studies, this research strategy encompasses bioassays, biochemical assays, and molecular biology, providing a comprehensive understanding of resistance mechanisms. However, the overall resistance phenotype is a complex combination of metabolic and target-site resistance [9]. One challenge in understanding insecticide resistance lies in the limitations of sequencing technologies, which often result in the separate analysis of metabolic and target-site resistance, preventing a holistic view of resistance mechanisms [129].

Earlier studies on insecticide resistance often focused on single genes, examining the relationship between mutations or changes in gene expression and resistance. However, resistance in pests is usually the combined result of enhanced detoxification enzyme activities and mutations in target genes [130]. Traditional approaches that treat metabolic and target-site resistance separately are insufficient for achieving a comprehensive understanding of resistance mechanisms [130], as they overlook the complexity of interactions between various genetic factors. The development of resistance in Lepidopteran pests is governed by intricate regulatory networks, which are crucial to uncover for effective pest management strategies [131]. Understanding these regulatory networks can provide valuable insights into the dynamic nature of resistance and identify potential molecular targets for intervention [8,14]. These insights deepen our understanding of the dynamic nature of resistance and highlight potential molecular targets for intervention in pest management strategies [132].

## 6. Future Prospects for the Applications of Functional Genomics Technologies

### 6.1. Emerging Gene-Editing Technologies and Their Potential Applications

Gene-editing technologies such as CRISPR/Cas9, TALENs, and the recently developed DNA- and RNA-targeting editing systems are reshaping insect gene research. The CRISPR/Cas9 system, with its simplicity, efficiency, and ability to target multiple gene loci simultaneously, has shown great potential in tackling issues related to Lepidopteran pest physiology, such as inhibiting reproduction, reducing survival, and counteracting insecticide resistance [17]. While CRISPR/Cas9 has gained popularity for its versatility, TALENs offer higher target specificity, which is beneficial in certain contexts, particularly for the precise manipulation of resistance and immunity studies [19]. Looking forward, these technologies may be utilized to edit resistance genes, control sex ratios, suppress reproduction, and establish gene drive systems, enhancing pest management efficiency [133]. A particularly promising avenue involves using CRISPR/Cas9 to induce sex imbalance, favoring male offspring to control pest populations effectively.

### 6.2. Future Direction of Gene Editing for Insect Physiology

A deeper understanding of the genomics and physiological pathways of Lepidopteran pests will enable a more targeted use of gene-editing technologies to regulate pest physiology. Future research may focus on the following areas: (1) Hormones and developmental genes: editing genes related to insect development, such as those involved in ecdysone and juvenile hormone, could disrupt pest growth, leading to developmental issues and reduced reproductive success. (2) Immune and metabolic regulation: modifying genes linked to immune function or metabolism could weaken pests’ resilience to environmental stressors, thereby reducing their survival. (3) Reproductive regulation: targeting reproductive genes could inhibit germ cell development or disrupt reproductive hormone signaling, thereby limiting pest populations. Such precise gene-editing strategies could offer species-specific solutions, minimizing risks to beneficial organisms.

### 6.3. Leveraging Genomic Data for Precision Resistance Management

With the growing availability of genomic data on Lepidopteran pests, precision management of insecticide resistance is becoming feasible. Key applications include: (1) Target resistance genes: analyzing resistance-associated mutations enables the use of gene-editing tools to knock out or modify these genes, restoring insecticide susceptibility. (2) Optimize pesticide use strategies: genomic insights can guide pesticide rotation plans, reducing the speed at which resistance develops. (3) Real-time resistance monitoring: functional genomics can aid in the development of tools to detect resistance mutations directly from field samples, allowing timely adjustments in pest control practices. Precision resistance management can thus enhance pesticide efficiency, minimize environmental impact, and promote sustainable pest control.

### 6.4. Role of Functional Genomics in Sustainable Pest Control

The application of functional genomics in sustainable pest management has long-term potential benefits, particularly in reducing reliance on chemical insecticides, by the following: (1) Enhancing biological control: genomic tools could optimize the effectiveness of natural enemies like parasitoid wasps or predatory insects by enhancing their predatory capacity or adaptability. (2) RNAi-based biopesticides: targeting pest-specific genes through RNA interference (RNAi) offers species-specific pest control options, reducing non-target effects and pesticide residues. (3) Insect pheromones and behavioral interference: genomic technologies could enable the development of behavioral control agents by modifying genes associated with sensory or behavioral responses, thereby reducing pests’ reproduction or foraging behaviors. Integrating functional genomics with ecology and biological control will support sustainable agriculture, especially as climate and environmental pressures increase.

Despite the revolutionary potential of CRISPR/Cas9, limitations remain. Off-target effects, limited efficiency in some species, and ethical and ecological concerns surrounding gene drives pose challenges. Moreover, regulatory and public acceptance hurdles must be addressed for broader application. Overcoming these limitations will be essential for the responsible and effective use of gene-editing technologies in pest management.

## 7. Conclusions

This study highlights the transformative potential of functional genomics in understanding and combating insecticide resistance in Lepidopteran pests. By focusing on resistance-related genes and pathways, functional genomics provides invaluable insights that contribute directly to developing targeted pest management approaches. The integration of gene-editing tools, transcriptomics, and bioinformatics allows for a comprehensive assessment of the genetic basis of resistance and the identification of key molecular targets for intervention, enabling the development of effective, species-specific control strategies.

The integration of genomic data with gene-editing technologies like CRISPR/Cas9 and TALENs marks a significant breakthrough to resistance management. These tools not only disrupt resistance mechanisms at the genetic level but also explore gene functions and gene–environment interactions, accelerating the development of innovative pest management solutions.

Looking ahead, functional genomics holds great promise for IPM strategies through precision-targeted interventions. The insights gained will empower IPM frameworks with tools for resistance prediction, early detection, and sustainable control solutions, improving agricultural productivity while minimizing the environmental impact.

In conclusion, functional genomics plays a key role in understanding insecticide resistance, providing actionable insights for resistance control. The combination of genomic data and gene-editing technologies offers a transformative avenue for resistance management, promising a balanced, sustainable path forward for IPM. However, further research is needed to address challenges in gene-editing technologies, such as off-target effects and species-specific variation, to fully realize their potential for precise and sustainable pest control.

## Figures and Tables

**Table 1:** Literature search strategy.

Databases	Boolean Operators	Numbers of Articles	Numbers of Screened Articles
WOS	TS = (“Lepidoptera” OR “lepidopteran”) AND TS = (“insecticide resistance” OR “pesticide resistance” OR “chemical resistance”) AND TS = (“RNA interference” OR “RNAi” OR “gene silencing” OR “dsRNA” OR “siRNA”)	102	74
Scopus	(TITLE-ABS-KEY (“Lepidoptera” OR “lepidopteran ”)) AND (TITLE-ABS-KEY (“insecticide resistance” OR “pesticide resistance” OR “chemical resistance”)) AND (TITLE-ABS-KEY (“RNA interference” OR RNAi OR “gene silencing” OR dsRNA OR siRNA))	56	
OS	TS = (“Lepidoptera” OR “lepidopteran”) AND TS = (“insecticide resistance” OR “pesticide resistance” OR “chemical resistance”) AND TS = (“GAL4/UAS” OR “TALEN” OR “CRISPR” OR “CRISPR/Cas9”)	31	21
Scopus	TITLE-ABS-KEY((“Lepidoptera” OR “lepidopteran”) AND (“insecticide resistance” OR “pesticide resistance” OR “chemical resistance”) AND (“GAL4/UAS” OR “TALEN” OR “CRISPR” OR “CRISPR/Cas9”))	30	

**Table 2:** Impact strength and level of genetic and toxicological effects.

Impact Type	Impact Standard	Description	Impact Strength	Impact Level
Effect_strength	EGI	Enhanced gene impact	2	High impact
	IRS	Impact on resistance or survival	1	Moderate impact
	IGET	Impact on gene expression and toxicity	0.5	Low impact
	IG	Impact on growth	0.3	Lower impact
Text_impact_strength	Increased Mortality	Mortality depending on dose	10	Extremely high impact
	Increased Toxicity	Significant increase in toxicity	8	High impact
	Increased Resistance	Increased resistance to drugs	6	Moderate impact
	Downregulated Gene Expression	Reduced gene expression, affecting resistance	5	Moderate impact
	Increased Sensitivity	Greater sensitivity to toxins or drugs	5	Moderate impact
	Reduced Growth and Tolerance	Impaired growth and reduced tolerance	3	Low impact
	Reduced Survival	Restored susceptibility to toxins	3	Low impact
	Increased Survival	Higher survival rate under stress	1	Lower impact
	Reduced Resistance	Decreased resistance to toxins or drugs	1	Lower impact
	Unrecognized	Effects that cannot be matched	0	Lower impact

**Table 3:** Application of TALENs and CRISPR/Cas9 technologies in resistance genes of Lepidopteran insects.

Species	Target Gene	Gene-Editing Technology	Gene Function	Reference
*B* *ombyx mori*	*ABCC2*	CRISPR/Cas9	Homozygous mutant resistant to Cry1Ac toxin, indicating its key role in resistance.	[105]
*B* *ombyx mori*	*ABCB1*	TALENs	Functions as a receptor for Cry toxins.	[106]
*Chilo suppressalis*	*RyR*	CRISPR/Cas9	Multiple point mutations confer diamide resistance.	[107]
*Helicoverpa armigera*	*HaCad*	CRISPR/Cas9	*HaCad* as a functional receptor of Cry1Ac.	[108]
*Helicoverpa armigera*	Cluster of nine *P450* genes	CRISPR/Cas9	Reduces survival when exposed to host plant chemicals and insecticides.	[109]
*Helicoverpa armigera*	*PgABCA2*	CRISPR/Cas9	Disruptive mutations increase resistance to Cry2Ab.	[110]
*Helicoverpa armigera*	*GST-119, GST-121*	CRISPR/Cas9	Knockout of the GST cluster using CRISPR/Cas9 significantly increased the sensitivity of the knockout strain to lambda-cyhalothrin.	[48]
*Helicoverpa zea*	*ABCC2*	CRISPR/Cas9	Mutations linked to Cry1Ac resistance ratios (7.3 to 39.8-fold).	[111]
*Helicoverpa zea*	*HzABCA2*	CRISPR/Cas9	Shows > 200-fold resistance to Cry2Ab compared to susceptible strain.	[112]
*Ostrinia furnacalis*	*OfABCC2*	CRISPR/Cas9	*Cry1Fa* resistance linked to 8 bp deletion mutation of *OfABCC2*.	[113]
*Ostrinia furnacalis*	*OfCad*	CRISPR/Cas9	Functions as a receptor for Cry1Ac; disruption confers moderate resistance.	[114]
*Plutella xylostella*	*Px* *ABCC2,* *Px* *ABCC3*	CRISPR/Cas9	*ABCC2* and *ABCC3* serve as midgut receptors for *Bt* Cry1 toxins.	[115]
*Plutella xylostella*	*nAChR α 6*	CRISPR/Cas9	*Px alpha 6* truncating mutation linked to high spinosyn resistance.	[116]
*Spodoptera exigua*	*SeRyRG4946E*	CRISPR/Cas9	Mutation confers high resistance to diamide insecticides.	[117]
*Spodoptera exigua*	*SeP-gp*	CRISPR/Cas9	Knockout increases susceptibility to abamectin and emamectin benzoate 3-fold but not to other insecticides.	[118]
*Spodoptera exigua*	*nAChR α 6*	CRISPR/Cas9	*Se alpha 6-KO* exhibits 373-fold higher resistance to spinosad.	[119]
*Spodoptera exigua*	*CYP9A186*	CRISPR/Cas9	Knockout restores susceptibility to EB, implicating the gene in avermectin resistance.	[120]
*Spodoptera exigua*	*nAChR α 6*	CRISPR/Cas9	*G275E* mutation confers high spinosyn resistance.	[121]
*Spodoptera exigua*	*CYP9A*	CRISPR/Cas9	*CYP9A* detoxifies plant defense compounds and insecticides.	[122]
*S* *podoptera exigua*	*nAChR α 1*	CRISPR/Cas9	*nAChR α 1*-KO shows increased resistance to various insecticides.	[123]
*Spodoptera frugiperda*	*SfABCC2*	CRISPR/Cas9	Acts as a functional Cry1F receptor.	[124]
*Spodoptera frugiperda*	*Vip3Aa*	CRISPR/Cas9	Knockout causes high resistance to *Vip3Aa* in fall armyworm and other pests.	[125]
*Spodoptera frugiperda*	*SfUGT50A15*	CRISPR/Cas9	*SfUGT50A15*-KO strain shows higher sensitivity to several insecticides than wild-type strains.	[126]

## Data Availability

No new data were created or analyzed in this study.

## Supplementary Materials

The following supporting information can be downloaded at https://www.mdpi.com/article/10.3390/ijms252212360/s1.
